# The Impact of Inadequate Temperature Storage Conditions on Aggregate and Particle Formation in Drugs Containing Tumor Necrosis Factor-Alpha Inhibitors

**DOI:** 10.1007/s11095-017-2341-x

**Published:** 2018-02-05

**Authors:** N. D. Vlieland, M. R. Nejadnik, H. Gardarsdottir, S. Romeijn, A. S. Sediq, M. L. Bouvy, A. C. G. Egberts, B. J. F. van den Bemt, W. Jiskoot

**Affiliations:** 1 Department of Clinical Pharmacy, Division Laboratory and Pharmacy, University Medical Center Utrecht, Heidelberglaan 100, 3584 CX Utrecht, The Netherlands; 2 Division of BioTherapeutics, Leiden Academic Centre for Drug Research, Leiden University, Leiden, The Netherlands; 3 Division of Pharmacoepidemiology and Clinical Pharmacology, Utrecht Institute for Pharmaceutical Sciences, Utrecht University, Utrecht, The Netherlands; 4 Department of Pharmacy, Sint Maartenskliniek, Nijmegen, The Netherlands; 5 Department of Pharmacy, Radboud Medical Center, Nijmegen, The Netherlands; 6 Department of Clinical Pharmacy and Toxicology, Maastricht University Medical Center, Maastricht, The Netherlands

**Keywords:** aggregation of antibodies, drug product characterization, freezing stress conditions, home storage, TNF-α inhibitors

## Abstract

**Purpose:**

To measure aggregate and particle formation in tumor necrosis factor-alpha (TNF-α) inhibitors etanercept, adalimumab and certolizumab pegol product samples after exposure to freezing temperature conditions similar to storage conditions previously observed in patients’ homes.

**Methods:**

TNF-α inhibitors in their original primary and secondary packaging were exposed to 32 freeze-thaw cycles (−10°C for 120min/5°C for 60 min) or continuous low storage temperature (−20°C for 96 h) before thawing at 2–8°C. Non-stressed products were used as controls. The products were analyzed by high pressure size exclusion chromatography (HP-SEC), dynamic light scattering (DLS), nanoparticle tracking analysis (NTA), micro-flow imaging (MFI) and second derivative ultraviolet (UV) spectroscopy.

**Results:**

Ten out of twenty-one stressed product samples (47.6%) showed increased particle numbers in the submicron and micron size range when compared to controls. For each product, DLS, MFI and NTA detected an increase in particle level in at least one stressed syringe (both continuous freezing and freeze-thaw), whereas HP-SEC and UV spectroscopy showed no differences between stressed and non-stressed products.

**Conclusion:**

TNF-α inhibitors are relatively resistant to freezing temperatures similar to storage conditions previously observed in patients’ homes. However, almost half of the stressed product samples showed formation of particles in the submicron and micron size range.

## Introduction

The introduction of drugs containing tumor necrosis factor-alpha (TNF-α) inhibitors has revolutionized treatments for many inflammatory diseases such as rheumatoid arthritis and inflammatory bowel disease [1]. TNF-α inhibitors, and other biologic drugs, differ from the traditional small molecule drugs as these are large complex proteins which are more prone to physical instability processes when exposed to external stress factors such as heat, freeze-thawing and agitation [2]. Due to the specific characteristics of biological drugs, these products need to comply with specific stability test programs and should be assessed regarding their potential immunogenicity [3,4]. According to the Summary of Product Characteristics documentation of TNF-α inhibitors, it is advised to store these products between 2°C and 8°C, not to expose them to freezing or agitation, and to protect them from light exposure [5,6].

A previous study showed that most patients do not store TNF-α inhibitors within this recommended temperature range; only 7% of patients were able to store TNF-α inhibitors continuously between 2 and 8°C [7]. Almost 25% of patients stored their TNF-α inhibitors below 0°C for 2 h or longer; 5.9% of patients stored their TNF-α inhibitors below 0°C for at least 24 h, with the lowest temperature measured around −20°C. In addition, almost 14% of the patients exposed their TNF-α inhibitors to at least three re-current freeze-thaw cycles with a median duration of almost 4 days. Six patients (2.4%) even exposed their drugs to at least 32 recurrent freeze-thaw cycles [7]. The most common consequence of exposing proteins to freezing temperature conditions is the formation of aggregates [8,9] which may lead to the development of antidrug antibodies and decreased drug effectiveness, as well as an increased probability of side effects [10,11].

Experimental data have shown that extreme low temperatures (−80°C) and multiple freeze-thaw cycles can induce formation of antibody aggregates in different non-commercial protein formulations [12,13]. However, it is unclear if marketed TNF-α inhibitors in their original formulation and primary container will undergo similar structural changes when exposed to less extreme low temperatures or multiple freeze-thaw cycles as observed in consumer refrigerators. The aim of this study was to assess aggregate and particle formation in TNF-α inhibitor product samples when exposed to temperature conditions similar to those observed in patients’ homes.

## Methods

### Materials

The following TNF-α inhibitors were kept in the original primary and secondary packaging and exposed to different temperature conditions as observed in the study by Vlieland *et al*. [7]: adalimumab 40 mg/0.8ml (six product samples Humira® A1-A5), certolizumab pegol 200 mg/ml (six product samples Cimzia® C1-C5), originator/biosimilar etanercept 50 mg/ml products (seven product samples Enbrel®(originator) E1-E6; six product samples Benepali® (biosimilar) B1-B5 (Table I). One package of adalimumab and certolizumab pegol contained two product syringes, packages of etanercept (originator and biosimilar) contained four product syringes. The tested TNF-α inhibitors have different characteristics: adalimumab is a human-derived recombinant monoclonal antibody, etanercept is a fusion protein (two TNF-α receptors and a human Fc fragment), certolizumab pegol is a pegylated anti-TNF-α antibody Fab’ fragment. We injected all (stressed and control) drug products from the prefilled syringe via the needle through the Teflon lined, pre-slitted screw caps into 1.5 mL sample vials, thereby mimicking as closely as possible a true injection by a patient. Prior to characterization, product samples were prepared with the following corresponding formulation buffers: etanercept: 10mg/ml sucrose, 5.8mg/ml NaCl, 5.3 mg/ml arginine, 3.9 mg/ml Na2HPO4.H2O, pH 6.3; adalimumab: 1.3 mg/ml citric acid, 1.5 mg/ml Na2HPO4.2H2O, 0.86 mg/ml NaH2PO4.2H2O, 12 mg/ml mannitol, 1 mg/ml polysorbate 80, 6.2 mg/ml NaCl, 0.3 mg/ml sodium citrate, pH 5.2; certolizumab: 0.28mg/ml (10mM) sodium acetate, 7.3 mg/ml (125 mM) NaCl, pH 4.7.

**Table I:** Product Sample Summary

Product	Strength	Volume	Lot nr.	Expiry date	Buffer	Control samples	Stressed samples	
Etanercept						2–8°C	Freeze-thaw	Continuous freezing
Enbrel^®^	50 mg	1.0 ml	N6158 N0062	12/2017 6/2018	10 mg/ml sucrose, 5.8 mg/ml NaCl, 5.3 mg/ml arginine, 3.9 mg/ml Na2HPO4.H2O, pH 6.3	1	3 (E1/E2/E3)	3 (E4/E5/E6)
Benepali^®^	50 mg	1.0 ml	CT0037 CT0026	9/2018		1	3 (B1/B2/B3)	2 (B4/B5)
Adalimumab								
Humira^®^	40 mg	0.8 ml	61145XD18	12/2017	1.3 mg/ml citric acid, 1.5 mg/ml Na2HPO4.2H2O, 0.86 mg/ml NaH2PO4.2H2O, 12 mg/ml mannitol, 1 mg/ml polysorbate 80, 6.2 mg/ml NaCl, 0.3 mg/ml sodium citrate, pH 5.2	1	3 (A1/A2/A3)	2 (A4/A5)
Certolizumab Pegol								
Cimzia^®^	200 mg	1.0 ml	195,843	9/2017	0.28 mg/ml (10 mM) sodium acetate, 7.3 mg/ml (125 mM) NaCl, pH 4.7	1	3 (C1/C2/C3)	2 (C4/C5)

### Applied Freezing Stress Conditions

**Fig. 1:**
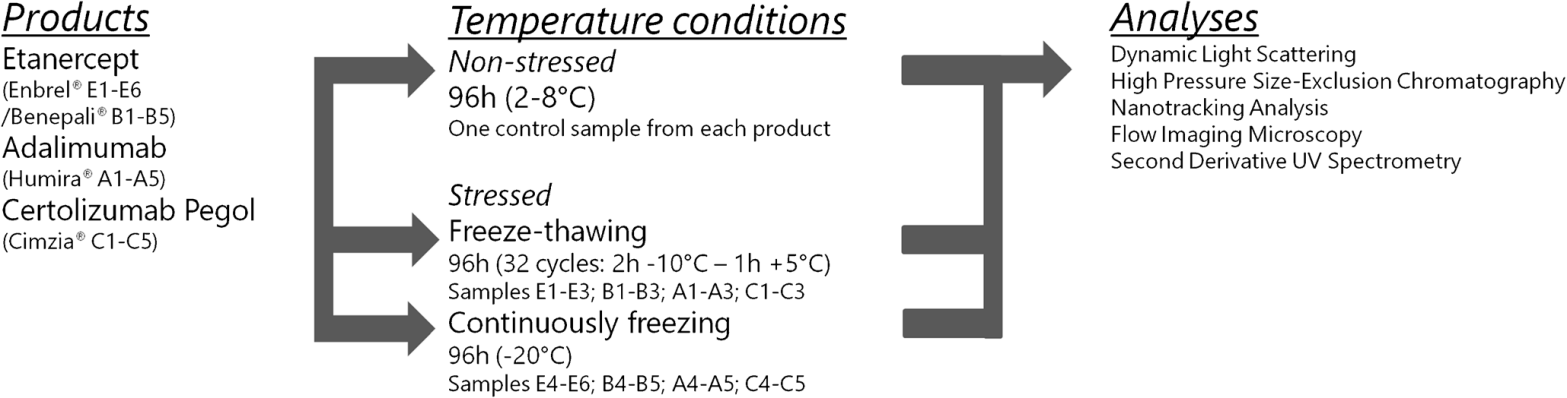
Overview experiments showing four different products, storage conditions and different analyses. h = hours.

Temperature conditions were simulated by usage of a Slow Programmable Freezer (Sylab Icecube 1810). This freezer makes use of liquid nitrogen and allows for applying storage temperatures between +5°C and −20°C in a reliable setting with little temperature variation (±0.5°C). TNF-α inhibitors were exposed to temperature conditions based on the lowest continuous temperature and recurrent freeze-thaw cycles observed in patients’ homes (Fig. 1) and subsequently tested for aggregate and particle formation. In the first stress protocol, three samples from each product (A1-A3; C1-C3; E1-E3; B1-B3) were exposed to multiple freeze-thaw cycles. Products were held at −10°C for 120min and subsequently thawed for 60 min at 5°C. This procedure was performed 32 times for a total exposure time of 96 h. Freezing/thawing speed for both stressing protocols was set to 1°C per minute. In the second stress protocol, samples from each product (A4-A5; C4-C5; B4-B5; E4-E6) were exposed to a continuous low storage temperature (−20°C) for a period of 96 h before thawing at refrigerator temperature (5°C). One sample from each product (stored in a refrigerator between 2 and 8°C) was used as control. All product samples were stored between 2 and 8°C before analysis.

### Product Characterization

The formation of aggregates and particles, and changes in protein conformation was determined by analyzing each stressed and non-stressed product with the methods described below.

#### Dynamic Light Scattering (DLS)

With DLS aggregates in the size range from about 1 nm to 1 μm can be detected. DLS was performed on a Malvern Zetasizer Nano (Malvern, Herrenberg Germany). 500 μl of the stressed and non-stressed product samples were analyzed in plastic cuvettes at 25°C using the automatic mode for identifying the best number of subruns and measurement time (*n* = 3). The Z-average diameter and polydispersity index (PdI) were calculated from the correlation function using the Dispersion Technology Software version 7.03 (Malvern, Herrenberg, Germany). All product samples were measured undiluted, except for the certolizumab products, which were diluted 4 fold with 0.28mg/ml (10mM) sodium acetate, 7.3 mg/ml (125 mM) NaCl, pH 4.7 due to the high viscosity of the product.

#### High Pressure Size Exclusion Chromatography (HP-SEC)

With HP-SEC the amount of monomers, dimers and fragments in the products can be detected and quantified. The non-stressed and stressed product samples were analyzed by HP-SEC, using a Yarra 3u SEC-2000 300 × 7.8mm (Phenomenex, Torrance, CA, USA) on an Agilent 1200 chromatography system (Agilent Technologies, Palo Alto, California) combined with a Wyatt Eclipse detector system (Wyatt Technology Europe GmbH, Dernbach, Germany), multi-angle laser light scattering (MALLS) detection with the DAWN® HELEOSTM (Wyatt Technology Europe GmbH) and at a flow rate of 0.5 ml/min. 5 μl of each diluted product sample was injected. All product samples were diluted with formulation buffer to a protein concentration of 1 mg/ml. The mobile phase was composed of 50 mM phosphate, 150 mM arginine and 0.025% NaN_3_ at pH 6.5. To quantify aggregation, UV absorption at 280 nm was recorded. From the MALLS signal, the root mean square (rms) diameter was calculated using the Berry Fit in the Astra software version 5.3.2.22 (Wyatt Technology Europe GmbH, Dernbach, Germany).

#### Nanoparticle Tracking Analysis (NTA)

Particles between 20 and 1000 nm can be detected with NTA. Measurements were performed with a NanoSight LM20, equipped with a sample chamber with a 640-nm laser operating at an angle of 173° with respect to the flow cell. All products were diluted with formulation buffer (Table I) to a protein concentration of 5 mg/ml. The product samples were injected into the chamber by an automatic pump (Harvard Apparatus, catalog no. 98–4362, Holliston, USA) using a sterile 1-ml syringe (BD Discardit II, Franklin Lakes, New Jersey). For each product a 90 s video was captured with the shutter set at 1495 and the gain at 400. Videos were analyzed by using the NTA 2.0 Build 127 software. The following settings were used for tracking of the particles: background extract on; brightness 0; gain 1.00; blur size 3 × 3; detection threshold 10, viscosity equal to that of water. All other parameters were set to the automatic adjustment mode.

#### Flow Imaging Microscopy

Micron sized particles up to 25 μm can be detected by MFI. A Micro-Flow Imaging (MFI) system (MFI5200, ProteinSimple, Santa Clara, USA), equipped with a silane coated flow cell (1.41 × 1.76 × 0.1 mm) and controlled by the MFI View System Software version 2, was used for flow imaging microscopy analysis. The system was flushed with 4 ml purified water at 6 ml/min prior to each measurement. The flow cell cleanliness was checked visually between measurements. The background was zeroed by flowing formulation buffer (Table I) and performing the ‘optimize illumination’ procedure. 0.3 ml of each product sample (undiluted, only certolizumab pegol was diluted fourfold due to high viscosity) without a pre-run volume because of the limited amount of product was analyzed at a flow rate of 0.17 ml/min and a fixed camera shot rate of 22 flashes per second. The data recorded by the system software was analyzed with MFI View Analysis Suite version 1.2. For each product, stuck, edge, and slow moving particles were removed by the software before data analysis. Because no pre-run volume could be used, the data was recorded from the start of the measurement until the product reached the flow cell. Therefore, data was processed in the time window from 0.7 to 1.7 min, in which the measurement was stable for all products. The equivalent circular diameter (ECD), which is the diameter of a circle that has an area equal to that of the particle imaged by MFI, was calculated and presented as a measure of the particle size (1–100 μm). Numbers of silicone oil droplet-like particles were calculated for each product (only for particles ≥5 um) by visual identification of typical oil droplets, which are round, have a smooth surface and are black with a small whitish spot in the center. In addition, we used the “find similar” procedure in the analysis software to identify particles that have image characteristics similar to those of the selected oil droplet-like particles [14].

#### Second Derivative UV Spectroscopy

Second derivative UV spectroscopy was used to detect conformational changes in the products upon stress. Measurements were performed using an Agilent 8453 UV–Vis spectrometer (Agilent Technologies, Waldbronn, Germany) according to the method described earlier (15). The product samples (diluted to 1 mg/ml) were measured in 2 ml half-micro quartz cuvettes (Hellma Benelux, Kruibeke, Belgium) with a path length of 10mm. The absorbance was measured from 240 to 340 nm with intervals of 1 nm using an integration time of 15 s. Background correction was performed with formulation buffer, diluted accordingly in freshly filtered Milli-Q grade water. The second derivatives of the spectra were calculated with UV–Visible ChemStation Software (Agilent Technologies, Walbronn, Germany) using a filter length of 9 nm and a polynomial degree of 4. Thereafter, the second derivatives were splined using 99 data points between the 1-nm measurement points. The vertical distance between the minimum at 283 nm and the maximum at 287 nm is denoted as ‘a’ and the vertical distance between the minimum and maximum at 290 and 295 nm as ‘b’ [15]. The ratio a/b is used to determine the exposure of tyrosine residues to bulk solvent, which is sensitive to changes in the tertiary structure.

## Results

### Temperature Stress Testing

All products were successfully exposed to the stress protocols mimicking multiple freeze-thaw cycles and continuous freezing temperatures.

### Product Characterization

#### Dynamic Light Scattering (DLS)

The Z-average diameter and PdI results for non-stressed and stressed products are summarized in Table II. Two product samples showed an increase in Z-average and PdI (product sample E3: Z-average 17.48 (SD 0.01)/PdI 0.27 (SD 0.01); product sample B3: Z-average 24.00 (SD 0.03)/PdI 0.27 (SD 0.03)) after multiple freeze-thaw stress conditions. In one certolizumab pegol product sample a difference in Z-average and PdI was detected after continuous freezing compared to the non-stressed product (C4: Z-average 10.13 (SD 0.02)/PdI 0.25 (SD 0.02)). Additional peaks in size distribution were detected after both stress conditions; product samples E2, E3, B3 exposed to multiple freeze-thaw stress conditions and product samples E4, C4, C5 exposed to continuous freezing stress conditions show peaks between 4000 nm and 6000 nm.

**Table II:** Second-Derivative UV Spectroscopy, DLS, NTA and HP-SEC Results for Etanercept (Originator and Biosimilar), Adalimumab and Certolizumab Pegol Drug Products Under Non-Stress Conditions (2–8 °C), Freeze-Thawing and Continuous Freezing Stress Conditions

Etanercept (originator)								
		Non-stressed 2–8 °C	Stressed (E1|E2|E3) 96 h FT − 10 °C/5 °C			Stressed (E4|E5|E6) 96 h − 20°C		
DLS	Z-average in nm (SD)	14.80 (0.01)	15.11 (0.00)	15.54 (0.01)	17.48 (0.01)	15.26 (0.01)	14.56 (0.00)	15.61 (0.02)
	PdI (SD)	0.11 (0.01)	0.09 (0.00)	0.14 (0.01)	0.27 (0.01)	0.01 (0.01)	0.00 (0.00)	0.02 (0.02)
HP-SEC	Monomer (%)	97.7	97.6	97.5	97.6	97.5	97.4	97.3
	Dimer (%)	2.3	2.4	2.5	2.4	2.5	2.6	2.7
	Molecular weight (Da) Monomer	1.3*10^5^	1.3*10^5^	1.3*10^5^	1.3*10^5^	1.3*10^5^	1.3*10^5^	1.3*10^5^
NTA (size estimation)	Mean in nm (SD)	259 (120)	181 (116)	203 (104)	246 (118)	335 (127)	339 (121)	363 (125)
UV spectroscopy	a/b ratio	0.96	0.96	0.96	0.96	0.97	0.93	0.96
Etanercept (biosimilar)								
		Non-stressed 2–8 °C	Stressed (B1|B2|B3) 96 h FT − 10 °C/5 °C			Stressed (B4|B5) 96 h − 20 °C		
DLS	Z-average in nm (SD)	14.82 (0.01)	15.05 (0.01)	14.80 (0.01)	24.00 (0.03)	14.80 (0.01)	14.91 (0.01)	
	PdI (SD)	0.06 (0.01)	0.09 (0.01)	0.05 (0.01)	0.27 (0.03)	0.08 (0.01)	0.07 (0.01)	
HP-SEC	Monomer (%)	97.9	97.9	97.9	97.4	97.9	98.0	
	Dimer (%)	2.1	2.1	2.1	2.6	2.1	2.0	
	Molecular weight (Da) Monomer	1.3*10^5^	1.3*10^5^	1.3*10^5^	1.3*10^5^	1.3*10^5^	1.3*10^5^	
NTA (size estimation)	Mean in nm (SD)	294 (151)	487 (99)	164 (86)	252 (119)	663 (345)	573 (261)	
UV spectroscopy	a/b ratio	0.96	0.95	0.96	0.96	0.96	0.97	
Adalimumab								
		Non-stressed 2–8 °C	Stressed (A1|A2|A2) 96 h FT − 10 °C/5 °C			Stressed (A4|A5) 96 h − 20 °C		
DLS	Z-average in nm (SD)	16.28 (0.01)	16.07 (0.01)	15.25 (0.01)	15.82 (0.01)	15.48 (0.01)	15.51 (0.01)	
	PdI (SD)	0.03 (0.01)	0.04 (0.01)	0.02 (0.01)	0.03 (0.01)	0.03 (0.01)	0.03 (0.01)	
HP-SEC	Monomer (%)	99.5	99.7	99.8	99.8	99.8	99.8	
	Dimer (%)	0.5	0.3	0.2	0.2	0.2	0.2	
	Molecular weight (Da) Monomer	1.6*10^5^	1.4*10^5^	1.4*10^5^	1.4*10^5^	1.5*10^5^	1.5*10^5^	
NTA (size estimation)	Mean in nm (SD)	328 (172)	252 (129)	205 (62)	281 (151)	246 (97)	325 (151)	
UV spectroscopy	a/b ratio	1.48	1.48	1.47	1.48	1.48	1.47	
Certolizumab pegol								
		Non-stressed 2–8 °C	Stressed (C1|C2|C3) 96 h FT − 10 °C/5 °C			Stressed (C4|C5) 96 h − 20 °C		
DLS	Z-average in nm (SD)	8.72 (0.01)	8.68 (0.02)	8.81 (0.00)	8.39 (0.01)	10.13 (0.02)	8.70 (0.01)	
	PdI (SD)	0.10 (0.02)	0.10 (0.02)	0.11 (0.00)	0.08 (0.01)	0.25 (0.02)	0.21 (0.01)	
HP-SEC	Monomer (%)	99.6	99.6	99.6	99.6	99.6	99.6	
	Dimer (%)	0.4	0.4	0.4	0.4	0.4	0.4	
	Molecular weight (Da) Monomer	5.7*10^4^	5.0*10^4^	5.0*10^4^	5.0*10^4^	5.8*10^4^	5.8*10^4^	
NTA (size estimation)	Mean in nm (SD)	527 (176)	304 (122)	398 (137)	335 (162)	415 (177)	455 (240)	
UV spectroscopy	a/b ratio	2.64	2.65	2.65	2.65	2.63	2.65	

#### High Pressure Size Exclusion Chromatography (HP-SEC)

**Fig. 2:**
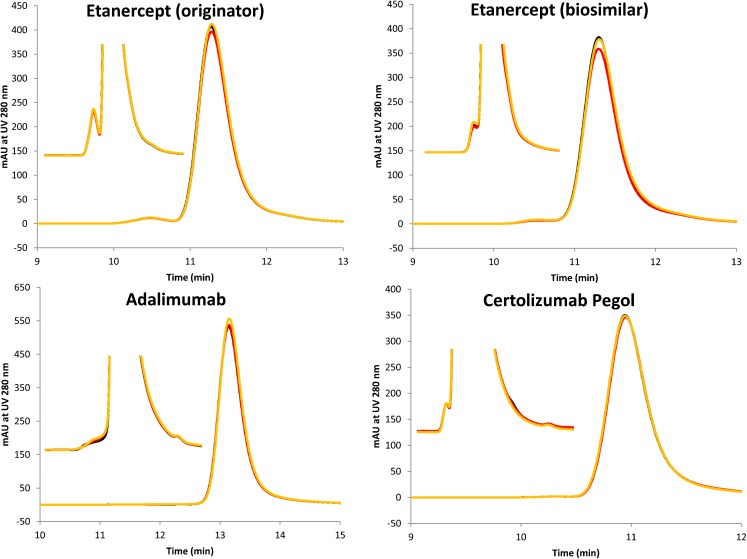
HP-SEC chromatograms. UV detection was performed at 280 nm. Graphs show controls *versus* two freezing stressed product samples. Black lines represent non-stressed product samples, red lines represent product samples exposed to freeze-thawing and orange lines represent product samples exposed to continue freezing stress conditions.

For the non-stressed drug products, monomer content was 97.7% for etanercept (originator), 97.9% for etanercept (biosimilar), 99.5% for adalimumab and 99.6% for certolizumab pegol (Table II). After both stress test conditions (multiple freeze-thawing and continuous freezing), monomer and dimer content for all drug products did not decrease compared to the non-stressed products (Fig. 2). Corresponding molecular weights, based on MALLS data, are presented in Table II for the main peak and correspond well with the expected molecular weights for the respective monomeric proteins.

#### Nanoparticle Tracking Analysis (NTA)

**Fig. 3:**
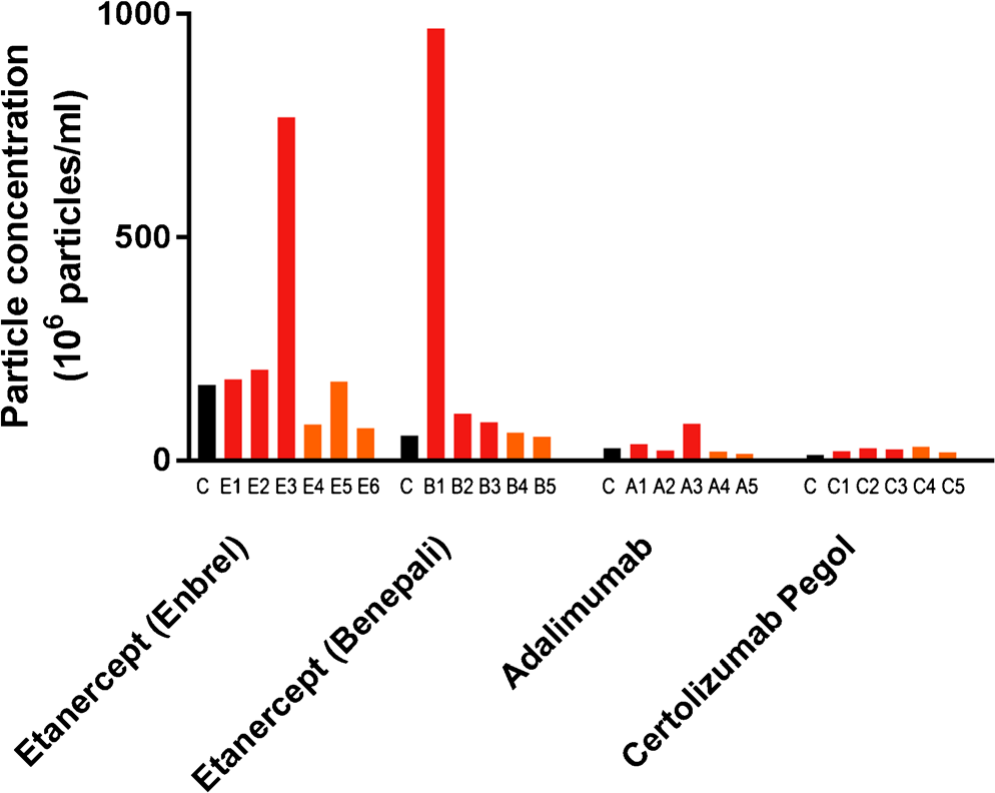
Nanoparticle tracking analysis (NTA). Black bars represent particle concentrations in non-stressed products (C = control sample). Red bars represent particle concentrations in products exposed to freeze-thaw stress conditions, Orange bars represent particle concentrations in products that were exposed to continuous freeze conditions.

For non-stressed products the following particle concentrations were detected: etanercept (originator) 1.7*10^8^ particles/ml, etanercept (biosimilar) 0.6*10^8^ particles/ml, adalimumab 0.3*10^8^ particles/ml, certolizumab pegol 0.1*10^8^ particles/ml. Two etanercept product samples showed an increase in particle concentration after multiple freeze-thaw cycles (product sample E3: 7.69*10^8^ particles/ml; product sample B1: 9.68*10^8^ particles/ml), which was not observed for the other products exposed to the same stress conditions or continuous freezing. No differences in particle concentrations were measured between non-stressed and stressed (both multiple freeze-thawing and continuous freezing) products of adalimumab and certolizumab pegol (Fig. 3). Changes in particle size were detected in etanercept (originator) and etanercept (biosimilar). Mean particle sizes for non-stressed product samples were 259 nm (SD 120) and 294 nm (151), respectively (Table II). Stressed samples showed larger mean particle sizes; E4: 335 nm (SD 127), E5: 339 (SD 121), E6: 363 (SD 125), B1: 487 nm (SD 99), B4: 663 nm (SD 345) and B5: 573 nm (SD 261).

#### Micro Flow Imaging (MFI)

**Fig. 4:**
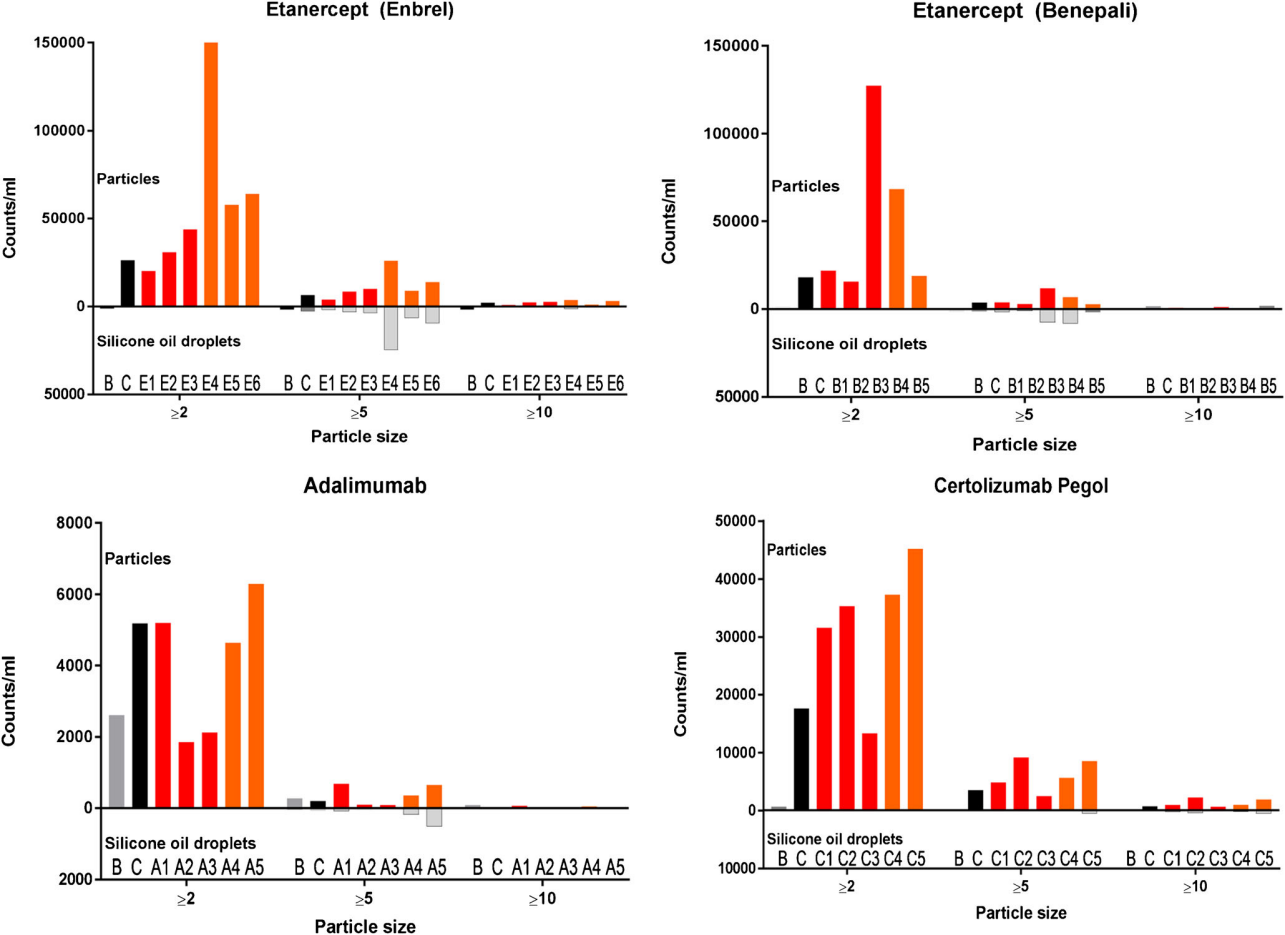
MFI results. Grey and black bars represent particle counts in buffer (**b**) and control products (**c**), respectively. Red bars represent particle counts products exposed to freeze-thaw stress conditions, orange bars represent particle counts in products that were exposed to continuous freeze conditions. Silicone oil droplet counts in different products are represented for particles ≥5 μm by light grey bars in the opposite direction.

**Fig. 5:**
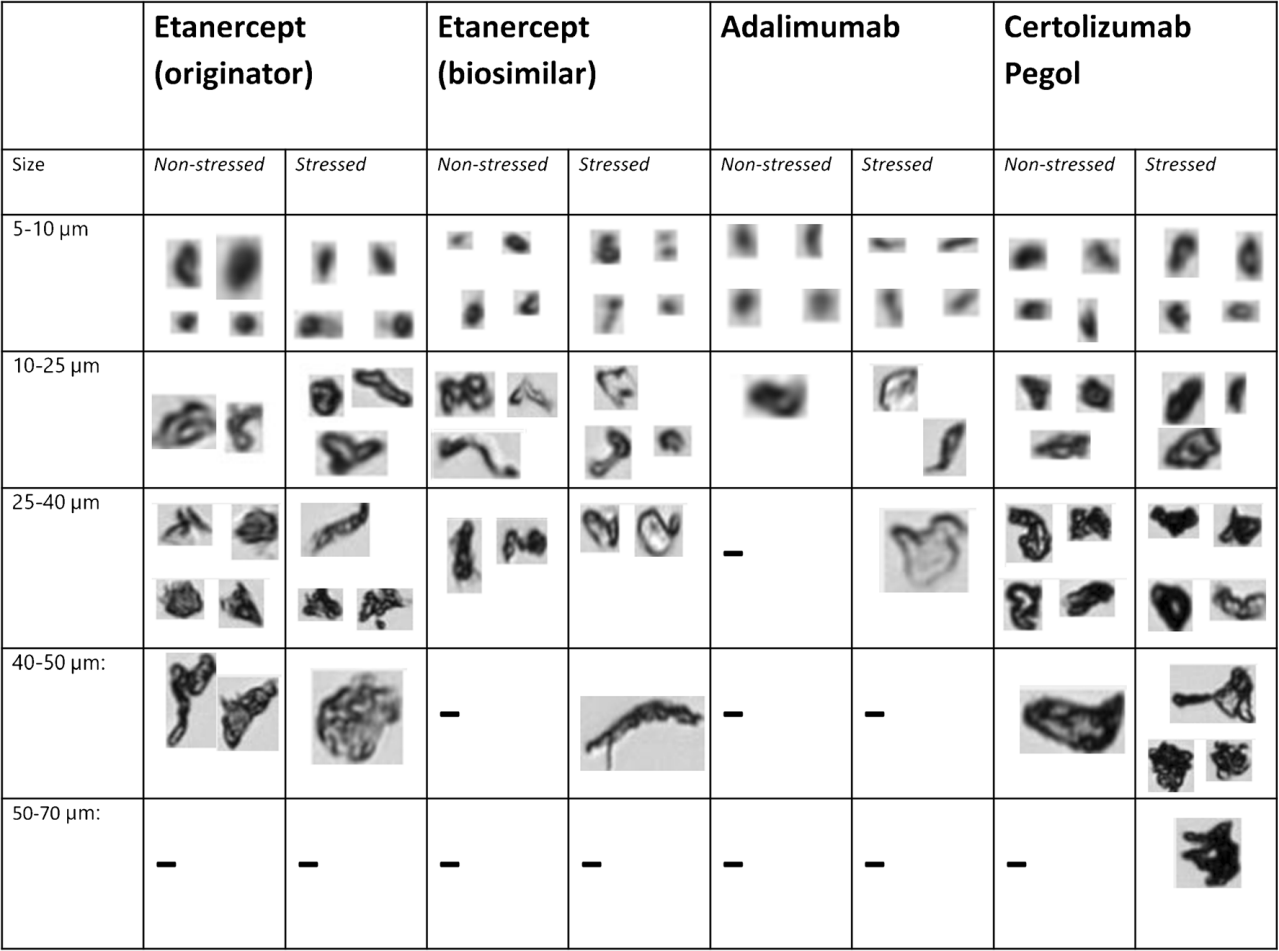
MFI results. Examples of MFI images for all products tested, stressed and non-stressed. Particle size ranges are shown in equivalent circular diameter (ECD). (−) = no particles in size range detected.

The concentrations of particles ≥2, ≥5, ≥10 and ≥25 μm are shown in Fig. 4. Representative images of particles are presented in Fig. 5. Non-stressed product sample for etanercept (originator) contained 26,308 particles ≥2 μm/ml and non-stressed product samples etanercept (biosimilar), adalimumab and certolizumab pegol contained respectively 18,168, 5193 and 17,640 particles/ml sized 2 μm or larger. Differences in particle concentrations were observed in etanercept products exposed to multiple freeze-thaw stress conditions: etanercept originator (product sample E3) and etanercept biosimilar (product sample B3). Certolizumab pegol products showed an increased particle concentration (C1, C2) after freeze-thaw stress conditions. Continuous freezing stress conditions also led to an increase in numbers of particles sized ≥2 μm in the following product samples: etanercept E4, E5, E6, B4, adalimumab product sample A5, certolizumab pegol product samples C4, C5.

Besides analyzing the total particle numbers, we used the “find similar” procedure of the MFI software to elucidate whether the increased particle numbers were due to silicone oil droplets, which could be released from the surface of the primary packaging materials, or to proteinaceous particles, or both. This distinction can be made for particles ≥5 μm based on morphological differences between silicone oil droplets and protein aggregates [14]. The results indicated that product samples (E4, E5, E6, B3, B4, A5) contained increased numbers of both silicone oil droplets and other, most likely proteinaceous particles. The percentage of silicone oil droplet-like particles in these product samples varied between 46% and 69% (for particles ≥5 μm; results not shown).

#### UV Spectroscopy

The a/b ratios for non-stressed etanercept (originator), etanercept (biosimilar), adalimumab and certolizumab pegol products were 0.96, 0.96, 1.48 and 2.64, respectively (Table I). No changes in a/b ratios between stressed (multiple freeze-thawing and continuous freezing) and non-stressed product samples were detected. Moreover, the peak positions for non-stressed product samples compared to stressed product samples (both multiple freeze-thawing and continuous freezing) were similar (results not shown).

### Results Summary

A summary of the results of all analytical methods used to detect and characterize aggregates and particles formed in the different stressed products is shown in Table III. In at least one sample of the four different products tested, to some extent more particles were detected compared to the non-stressed sample. Particles in the submicron and micron size range were detected in ten of the twenty-one TNF-α inhibitor product samples (47.6%), six product samples upon exposure to multiple freeze-thawing and four product samples after exposure to continuous freezing conditions. With HP-SEC and UV spectroscopy no differences in aggregate formation were detected between stressed (both multiple freeze-thawing and continuous freezing) and non-stressed products. With DLS, differences in aggregate level between one product sample exposed to multiple freeze-thawing and the non-stressed product sample were detected in etanercept originator and biosimilar products. After continuous freezing stress conditions, two etanercept (originator and biosimilar) product samples and certolizumab pegol product sample showed a higher Z-average compared to the non-stressed product. NTA testing showed differences in particle concentration in two stressed etanercept product samples (one originator/one biosimilar) upon freeze-thaw stress conditions compared to the non-stressed products. This result corresponds partially with DLS, for etanercept (originator) product sample E3, where both methods detect increased aggregate levels. Larger particles (>1 μm) were also detected with MFI: etanercept (originator and biosimilar) and certolizumab pegol showed an increased number of particles after freeze-thaw stress conditions. After continuous freezing stress conditions, in at least one product of etanercept (originator/biosimilar), adalimumab and certolizumab pegol an increase in the number of large particles was detected.

**Table III:** Overview of Product Characterization Experiments, Freezing Stress Conditions and Product Samples in Which Aggregates Were Detected

Detection technique	Detection range	Etanercept (originator)		Etanercept (biosimilar)		Adalimumab		Certolizumab pegol	
		96 h FT −10 °C/5 °C	96 h −20 °C	96 h FT −10 °C/5 °C	96 h −20 °C	96 h FT −10°C/5°C	96 h −20°C	96 h FT −10°C/5°C	96 h −20°C
DLS	Size range: <1 μm	+	0	+	0	0	0	0	+
HP-SEC	Relative amount mono−/dimer/fragments	0	0	0	0	0	0	0	0
NTA	Size range: <1 μm	+	0	+	0	0	0	0	0
MFI	Size range: >2 μm	+	+	+	+	0	+	+	++
UV spectro-scopy	Structural changes	0	0	0	0	0	0	0	0

*FT Products exposed to freeze-thaw stress (96 h)*

*CF Products exposed to continue freeze stress (96 h)*

*0 no differences in aggregate/particle level in stressed* vs *unstressed products*

*+ higher aggregate/particle levels in at least 1 stressed* vs *unstressed product*

*++ higher aggregate levels in stressed* vs *unstressed products*

## Discussion

This study shows that temperature conditions similar to those that occur in patients’ homes have minor impact on the level of aggregates and particles in product samples of etanercept (Enbrel® and Benepali®), adalimumab (Humira®) and certolizumab pegol (Cimzia®). Nevertheless, products exposed to these temperature conditions contained more particles in the submicron and micron size range. Almost half of the product samples which were exposed to multiple freezing stress conditions (47.6%; six freeze-thawing and four continuous freezing) showed larger numbers of subvisible particles (>1 μm) compared to non-stressed products.

Our results are qualitatively in line with other studies investigating the formation of aggregates in IgG antibody formulations after exposure to freezing stress conditions, which describe the formation of few aggregates >1 μm [13,16]. Although others have observed changes in monomer/dimer/oligomer content with HP-SEC and conformational changes with UV spectroscopy [12,17], we did not find such changes after exposing TNF-α inhibitors to freezing stress conditions. Moreover, not all product samples showed elevated particle levels. For those product samples that did show elevated particle levels by NTA and/or MFI, HP-SEC results indicate that these particles corresponded to a minute fraction of the total amount of protein. This low level of protein aggregation may be due to the fact that in the current study we used marketed products in their original formulation and primary container, whereas the cited studies were done on non-commercial IgG molecules. Moreover, the stress conditions applied in our study were relatively mild when compared to the other studies. With DLS, three product samples (E3, B3, C4) showed an increase in aggregate level after freezing stress conditions. These results were partially in line with the NTA data, showing the formation of particles in one etanercept sample (E3) after multiple freeze-thawing, but not for etanercept (biosimilar) samples and certolizumab pegol. MFI data showed the formation of large aggregates (>1 μm) in at least one sample of all products after both stress conditions (E3, E4, B3, B4, A5, C1, C2, C4, C5), except for adalimumab upon multiple freeze-thawing. In addition, an increase in the number of silicone oil droplets was detected with MFI in some product samples (E4, E5, E6, B3, B4, A5) with the percentages of silicone oil droplets ranging between 46 and 69% [14].

Freeze-thawing has been described as having a smaller impact on the stability of biologics compared to heating or agitation and shows the formation of only few aggregates in the micron and submicron size range [12]. Our observations confirm other findings suggesting that the level of aggregation upon freeze-thaw stress is generally low with particles in the low micron-size range as main degradation product [12,17–19]. In this study products were exposed to two stress conditions: multiple freeze-thawing and continuous freezing. Although one would expect that multiple freeze-thawing cycles would have more impact than continuous freezing stress, we did not observe such an effect. Subjecting products to continuous low temperatures might increase ice crystal formation or ice texture changes in some of the products, thereby increasing aggregation [20].

In theory, exposing products to inadequate storage conditions as previously reported could induce the formation of aggregates which could lead to the development of antidrug antibodies and might subsequently affect treatment outcome [10]. Although recent studies have shown that home storage conditions for TNF-α inhibitors are often not adequate [7], there is no evidence that this has resulted in the development of antidrug antibodies or has had other clinical consequences for patients. The relation between inadequate storage, protein aggregation and immunogenicity has not been investigated in humans due to ethical reasons, but a number of experiments in animal models have shown that the amount, size, and nature of aggregates to a certain extent determines the immunogenic potential of a protein drug [10,19]. A recent post marketing study on peginesatide (an erythropoiesis-stimulating agent) in relation to the occurrence of severe adverse events (49 cases of anaphylaxis, including 7 fatalities) linked these events to a higher concentration of subvisible particles [21]. A prospective study would be needed to investigate the complex relation between storage conditions of TNF-α inhibitors, aggregate formation, immunogenicity and therapy outcomes.

In this study, there were limitations concerning the number of different TNF-α inhibitor products and the number of samples from each product that could be tested. The availability of more samples and products for testing might have enabled us to get a better and more reliable assessment of aggregation risk for different biological drugs that are not stored according to label instructions. We only stressed products for 96 h, whereas patients store products in their refrigerator for up to three months. This difference might have resulted in an underestimation of the number of products that contained aggregates after freezing stress conditions. Extending the stress period would give a better estimation how TNF-α inhibitor products can change during home storage. In addition, we did not assess other important stress conditions that TNF-α inhibitors may be exposed to during transport and long storage periods, such as agitation and light exposure. Exposure to conditions outside the recommended storage conditions might also affect container closure integrity of the drug product, which can have impact on its stability and sterility. As this is one of the first studies in its kind, more research is required in order to investigate the consequences of inadequate storage for product quality and its effect on immunogenicity and clinical response on treatment with TNF-α inhibitors.

## Conclusion

The studied TNF-α inhibitors remain relatively stable with regard to the number of aggregates and particles when exposed to temperature storage conditions seen at patients’ homes. However, aggregation as a result of freezing stress conditions appears to be probabilistic, as we detected subvisible particles (>1 μm) in almost half of the product samples. Low temperatures (−20°C) and multiple freeze-thaw cycles as observed in consumer refrigerators can induce the formation of few aggregates in different TNF-α inhibitor products.

## Abbreviations

DLS: Dynamic light scattering
ECD: Equivalent circular diameter
HP-SEC: High pressure size exclusion chromatography
MALLS: Multi angle laser light scattering
MFI: Micro-Flow Imaging
NTA: Nanoparticle tracking analysis
PdI: Polydispersity index
TNF-α: Tumor necrosis factor-alpha
UV: Ultraviolet light
